# Modeling the relationships among student-perceived teacher support, academic buoyancy, and L2 engagement: a mixed-methods approach

**DOI:** 10.3389/fpsyg.2026.1795937

**Published:** 2026-06-12

**Authors:** Xiaojing Hu, Shujun Tong, Jingke Xu

**Affiliations:** School of Education, Xi'an International Studies University, Xi'an, China

**Keywords:** a mixed-methods approach, academic buoyancy, EFL learners, L2 engagement, teacher support

## Abstract

**Introduction:**

The present study aims at investigating the relationship between student-perceived teacher support and their L2 engagement through the mediating role of academic buoyancy in EFL contexts.

**Methods:**

In doing so, a group of 415 Chinese EFL learners were recruited to complete three closed-ended questionnaires, assessing the levels of teacher support, academic buoyancy and L2 engagement. Besides, 48 students were invited to respond to an open-ended questionnaire to triangulate the quantitative findings and gain a nuanced understanding of the interrelationships among these constructs.

**Results:**

Correlation analysis indicated that student-perceived teacher support, academic buoyancy and L2 engagement were positively and significantly correlated with each other. Structural equation modeling analysis indicated a significantly positive relationship between EFL learners’ perceptions of teacher support and L2 engagement. In addition, academic buoyancy partially mediated the relationship between teacher support and L2 engagement. The qualitative data have triangulated the quantitative findings and corroborated the impact of student-perceived teacher support on enhancing EFL learners’ academic buoyancy and L2 engagement.

**Discussion:**

This paper has confirmed the essential role of teacher support in developing EFL learners’ ability to cope with setbacks and pressures as well as enhancing their involvement in the ordinary course of English learning.

## 1 Introduction

Initially, the construct of engagement was defined as the extent of students’ active participation in academic tasks, aiming at exploring the underlying mechanisms of student disengagement and educational attainment (Jimerson et al., 2003). Besides their involvement in academic tasks, social–emotional and behavioral engagement should also be included in students’ goals of schooling (Christenson et al., 2012). Thus, recent researchers generally acknowledge that engagement is a multidimensional construct rather than being viewed solely as students’ academic attributes (Fredricks et al., 2004; Reeve and Tseng, 2011). A large body of empirical evidence has repeatedly identified engagement as a predictor of favorable educational outcomes, including the improvement of academic achievement (e.g., Hiver et al., 2024; Reinke et al., 2022), the decrease of drop-out rate (Archambault et al., 2022; Sosa-Alonso et al., 2025; Klem and Connell, 2004), and the reduction of high-risk behaviors (e.g., Christenson et al., 2012; Reschly and Christenson, 2022). Moreover, engagement may contribute to students’ mental health and psychological well-being (Schwinger et al., 2014; Wong et al., 2024). Engagement is particularly critical in language learning contexts, given that mastering a new language constitutes a complex and prolonged undertaking characterized by multiple potential challenges that demand sustained learner engagement (Zou et al., 2025a).

As a key social factor in educational contexts, teacher support exerts a crucial influence on students’ learning processes and outcomes. “Successful learning may be very difficult, if not impossible” when teachers fail to provide sufficient support to students (Piechurska-Kuciel, 2011, p. 84). The influence of teacher support on students’ L2 engagement has been well established in the traditional EFL contexts (e.g., Chen, 2025; Sadoughi and Hejazi, 2021, 2023; Zhang and Wang, 2023) and in the GenAI-mediated language learning contexts (e.g., Wang and Zou, 2026). Nevertheless, the potential mediating role of academic buoyancy in the relationship between student-perceived teacher support and L2 engagement remains unexplored in prior research. Academic buoyancy deals with students’ abilities to cope with setbacks and challenges in their regular school life (Martin and Marsh, 2008). As a positive personality factor, academic buoyancy enables students to maintain high motivation and engagement even when they encounter difficulties and challenges in learning (Collie et al., 2015). Besides, extant research on the relationship between perceived teacher support and engagement has relied heavily on quantitative designs. Scholars are thus encouraged to adopt additional methodological lenses to achieve a more nuanced understanding of this interplay (Prananto et al., 2025). In order to address these gaps, the current study uses a mixed-methods design to map the interplay of student-perceived teacher support, academic buoyancy, and L2 engagement among Chinese secondary-level EFL learners. The research findings are anticipated to inform targeted interventions and scaffolding frameworks to bolster EFL learners’ academic buoyancy and L2 engagement. The theoretical rationale and empirical evidence for academic buoyancy as a potential mediator in the relationship between teacher support and L2 engagement will be discussed in the following section.

## 2 Literature review

### 2.1 Theoretical framework

The rationale for studying the interrelationships of the constructs included in the current study is based on Self-Determination Theory (SDT), which has been theoretically validated as a lens for elucidating individuals’ motivated behaviors across diverse contexts (Ryan and Deci, 2020). According to the SDT, when people’s basic psychological needs, namely autonomy, competence, and relatedness, are satisfied, these need-satisfactions function as inherent catalysts for personal growth, social integration, and psychological well-being (Ryan and Deci, 2017). In school contexts, the fulfillment of learners’ basic psychological needs may vitalize learners’ intrinsic motivation, thereby bolstering their academic engagement (Niemiec and Ryan, 2009; Ryan and Deci, 2020). The contextual factors that support learners’ fundamental psychological needs for autonomy, competence and relatedness play a significant role in influencing student motivation and academic engagement, which in turn contribute to their academic achievement at school (Wang and Holcombe, 2010). Teachers are generally known as the essential contextual factor in language teaching and learning (Sadoughi and Hejazi, 2022). Teacher support bolsters learners’ self-efficacy (Hughes and Chen, 2011; Ma, 2025) and simultaneously cultivates the interest, enthusiasm, persistence, and effort that collectively promote their engagement in language learning (Roorda et al., 2011). Based on the SDT, we hypothesize that sufficient support provided by teachers may satisfy students’ basic psychological needs, which empowers them to cope with difficulties in the mundane practices that constitute everyday school experience, and in turn strengthens their engagement in learning tasks and activities.

### 2.2 L2 engagement

Engagement originates within the domain of educational psychology, which is conceptualized as “the quality of a student’s connection or involvement with the endeavor of schooling and hence with the people, activities, goals, values and place that compose it” (Skinner et al., 2009, p. 494). When this construct is relocated to the second-language classroom, it is reframed as L2 engagement: learners’ efforts in the process of acquiring a second or foreign language (Hiver et al., 2021). As for its dimensions, most researchers generally describe it as a multifaceted construct including behavioral, emotional and cognitive dimensions (e.g., Fredricks et al., 2004; Jimerson and Chen, 2022). Behavioral engagement is concerned with students’ involvement of observable and tangible attention, effort and persistence in a learning activity (Fredricks et al., 2019). Emotional engagement reflects learners’ experience of favorable emotions, such as interest or enjoyment, alongside the absence of unfavorable emotions in doing tasks and activities (Reeve, 2013). Cognitive engagement is characterized by learners’ deep psychological commitment and their deployment of self-regulatory or strategic approaches in learning (Skinner et al., 2009). In the recent decade, Reeve and Tseng (2011, p. 258) adds agentic engagement, defined as “students’ constructive contribution into the flow of the instruction they receive” (e.g., sharing their preferences with language teachers and offering suggestions to improve teaching voluntarily), to the conventional three dimensions. The four dimensions are highly correlated and can measure EFL learners’ engagement levels effectively (Oga-Baldwin, 2019).

Given its broad positive impact on educational outcomes, engagement merits closer examination of its antecedents. In general education, instructional quality, teacher support and teacher-student rapport have been documented as antecedents of student engagement (e.g., Hofkens and Pianta, 2022; Liu et al., 2017; Reinke et al., 2022). In L2 contexts, self-efficacy, teacher communication behaviors (e.g., teacher credibility, teacher confirmation and teacher care), growth language mindset, grit, and well-being are found to be the predictive factors of EFL learners’ engagement (Derakhshan et al., 2022; Derakhshan and Fathi, 2025; Wang and Kruk, 2024). Empirical studies have also demonstrated that perceived teacher support boosts learner engagement in traditional EFL settings and in the GenAI-mediated language learning contexts (e.g., Chen, 2025; Sadoughi and Hejazi, 2021, 2023; Wang and Zou, 2026; Zhang and Wang, 2023). Nevertheless, the pathways through which teacher support influences L2 engagement remain elusive. Moreover, previous studies on the relationship between teacher support and L2 engagement predominantly employ a quantitative design. The present study addresses these gaps by examining how teacher support relates to EFL learners’ L2 engagement, with academic buoyancy hypothesized as a partial mediator. Both quantitative and qualitative data will be utilized to illuminate this mechanism.

### 2.3 Teacher support

As a main force of social support in the school settings and an important aspect of classroom environment, teacher support is widely investigated in general education (for a review, see Prananto et al., 2025). In L2 settings, teacher support is defined as “teachers’ assistance with students’ language learning”, which includes three dimensions, namely emotional, instrumental and academic support (Liu and Li, 2023, p. 3). Emotional support pertains to L2 learners’ perceptions that their language teachers love them, encourage them, care for them and trust them (Sadoughi and Hejazi, 2022). Instrumental support is concerned with practical and tangible support such as time or skills when students confront demanding activities (Semmer et al., 2008). Academic support, by contrast, encompasses the transmission of domain-specific knowledge and the delivery of formative feedback by language teachers to scaffold second or foreign language development (Liu and Li, 2023).

As confirmed by numerous studies, student-perceived teacher support has been documented to facilitate learners’ willingness to communicate (Han and Ganapathy, 2025; Piechurska-Kuciel, 2015; Wei and Xu, 2022), strengthen their self-efficacy beliefs regarding language-learning competence (Ma, 2025), and foster the perseverance and coping resources required to navigate academic adversities (Fu, 2024; Li et al., 2025; Sadoughi and Hejazi, 2023). For instance, Ma (2025) indicated that emotional support and instrumental support could contribute to the enhancement of EFL learners’ self-efficacy beliefs, thereby increasing their willingness to communicate among 4,760 secondary school students. Using a quantitative design, Fu (2024) showed that teacher support could contribute to EFL learners’ academic buoyancy in language learning among a sample of 1955 EFL learners.

Teacher support has also been found to act as a catalyst for students’ motivation (e.g., Derakhshan et al., 2025; Ou, 2025), while mitigating learners’ negative emotions—such as anxiety, burnout, and boredom—when learning a foreign language (e.g., Jin et al., 2017; Liu et al., 2023). Derakhshan et al. (2025) showed a significantly strong and positive relationship between teacher support and reading motivation. Additionally, the findings demonstrated that more than 50% of the variance in L2 reading motivation was explained by teacher support and its indirect effects via learner emotions (i.e., enjoyment, anxiety and boredom). In another study, Liu et al. (2023) found that students’ perceived teacher support was negatively associated with foreign language anxiety among 1,401 Chinese high school students.

Moreover, teacher support has been empirically identified as a pivotal antecedent of L2 engagement (Chen, 2025; Sadoughi and Hejazi, 2021, 2023; Zhang and Wang, 2023) and language achievement (e.g., Derakhshan and Fathi, 2025; Li et al., 2023; Piechurska-Kuciel, 2013). In a recent study, Derakhshan and Fathi (2025) found that teacher support was positively and significantly associated with EFL learners’ academic achievement. Moreover, academic buoyancy and L2 boredom sequentially mediated the relationship between perceived teacher support and foreign language achievement. In another study conducted by Sadoughi and Hejazi (2023), teacher support was found to be a predictor of academic engagement through the mediating role of L2 grit among 295 high school and undergraduate Iranian EFL learners.

### 2.4 Academic buoyancy as a potential mediator

Academic buoyancy is first suggested by Martin and Marsh (2008, p. 53), conceptualized as “students’ ability to successfully deal with academic setbacks and challenges that are typical of the ordinary course of school life”. Unlike academic resilience, which addresses learners’ ability to endure severe or persistent adversities viewed as significant threats to the developmental processes, academic buoyancy focuses on managing the everyday setbacks and pressures inherent in routine academic life (Martin and Marsh, 2009). Extending this construct to L2 settings, Yun et al. (2018, p. 1) characterizes L2 academic buoyancy as “learners’ capacity to overcome the setbacks, challenges, and pressures that are part of the ordinary course of school life for instructed L2 learning”. A substantial body of empirical research has established that academic buoyancy serves as a significant predictor of students’ academic achievement across disciplines (e.g., Datu and Yang, 2021; Li et al., 2023; Miller et al., 2013; Putwain et al., 2015; Weissenfels et al., 2022; Yun et al., 2018).

As outlined in the theoretical framework, the SDT offers the rationale for treating academic buoyancy as a mediator. Empirical evidence also suggests that academic buoyancy may function as an underlying mediator through which students’ perceived teacher support translates into academic engagement. Several studies have showed that teacher support plays a contributing role in enhancing learners’ academic buoyancy in both general education and in L2 settings (Derakhshan and Fathi, 2025; Fu, 2024; Granziera et al., 2022; Li et al., 2025; Pitzer and Skinner, 2016). For instance, a longitudinal study carried out by Pitzer and Skinner (2016) revealed that teacher support could help students with low levels of academic buoyancy to develop their abilities to handle academic difficulties and setbacks. Students who experienced reduced teacher support demonstrated diminished levels of academic buoyancy. Granziera et al. (2022) further demonstrated that instrumental support perceived by students covaried with academic buoyancy among Singaporean secondary students and Australian primary pupils. Within L2 settings, Li et al. (2025) reported that each facet of perceived teacher support exerted a positive influence on EFL learners’ academic buoyancy.

**Figure 1:**
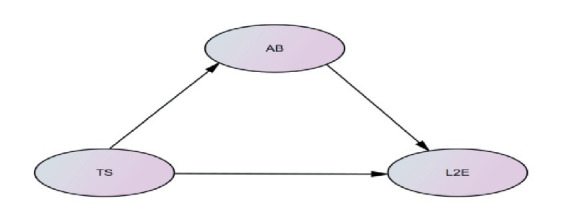
The proposed structural framework. TS refers to teacher support; AB refers to academic buoyancy; L2E refers to L2 engagement.

In addition, a positive linkage is found between academic buoyancy and academic engagement in both general education and in L2 settings (e.g., Datu and Yang, 2016; Thomas and Allen, 2021; Ursin et al., 2021; Wang, 2024). For instance, Datu and Yang (2016) found that academic buoyancy was positively correlated with both behavioral and emotional engagement in a sample of 402 university students in the Philippines. Wang (2024) showed that academic buoyancy could contribute to the enhancement of L2 engagement among Chinese secondary-level EFL learners. Based on these findings, we hypothesize that academic buoyancy may mediate the relationship between student-perceived teacher support and L2 engagement. Figure 1 shows the hypothesized model.

*Hypothesis 1:* There is a significant positive relationship between teacher support and L2 engagement.

*Hypothesis 2:* Academic buoyancy mediates the relationship between teacher support and L2 engagement.

## 3 Methodology

### 3.1 Participants

Participants comprised 415 students enrolled in a senior high school located in Shandong Province in China. There were 156 first-year EFL learners (37.6%), 162 s-year EFL learners (39%) and 97 third-year EFL learners (23.4%). The sample consisted of 203 males (48.92%) and 212 females (51.08%) with an age range of 15 to 18 years old. Most of them started to learn English at Grade Three in the primary school, and they had no overseas learning experiences. In the quantitative phase, all participants completed three closed-ended questionnaires. In the qualitative phase, 48 students from the initial sample completed an open-ended questionnaire.

### 3.2 Measures

#### 3.2.1 Student-perceived teacher support questionnaire (TSQ)

Student-perceived teacher support was assessed using Liu and Li (2023), which was a six-point Likert scale with three sub-scales: academic support (5 items; e.g., The English teacher imparts practical knowledge to us (such as sentence patterns, etc.), instrumental support (3 items; e.g., The English teacher helps me choose suitable extra-curricular reading materials) and emotional support (4 items; e.g., The English teacher understands the difficulty of my English learning). This scale was validated by Liu and Li (2023) among a group of 1,401 Chinese EFL learners (Cronbach’s *α* = 0.893). In the present study, an item in the subscale of emotional support was excluded because its factor loading was lower than 0.4 (Stevens, 1992), and Cronbach’s alpha for the overall scale was 0.858.

#### 3.2.2 Academic buoyancy questionnaire (ABQ)

EFL learners’ academic buoyancy was assessed using Yun et al. (2018). It had 46-point Likert-scale items (e.g., When I run into a difficult problem in English language class, I keep working at it until I think I’ve solved it) and was validated among a group of 305 Chinese university EFL learners by Wang (2024) (Cronbach’s α = 0.877). In the present study, Cronbach’s alpha for the overall scale was 0.907.

#### 3.2.3 L2 engagement questionnaire (L2EQ)

L2 engagement questionnaire was adopted from Reeve and Tseng (2011). The items were contextualized by inserting the phrase “in English class”. It was a seven-point Likert scale, which had 22 items in four sub-scales: agentic engagement (5 items, e.g., During English class, I ask questions), behavioral engagement (5 items, e.g., I listen carefully in English class), emotional engagement (4 items, e.g., When I am in English class, I feel curious about what we are learning) and cognitive engagement (8 items, e.g., When doing school work, I try to relate what I’m learning to what I already know). The scale was validated by a group of 642 Chinese non-English major EFL learners in Zhang and Wang (2023) (Cronbach’s α = 0.79). In the present study, the internal consistency of the scale was excellent, with Cronbach’s alpha of 0.959 for the total scale.

#### 3.2.4 Open-ended questionnaire

Besides the closed-ended questionnaires, an open-ended questionnaire was developed to triangulate the quantitative findings and attain more nuanced and comprehensive information about the interrelationships of the constructs under investigation. Four questions were employed to collect the qualitative data. The participants were asked to write open-ended responses about the interrelationships among teacher support, academic buoyancy, and L2 engagement. Two professors in applied linguistics evaluated four questions for content adequacy and linguistic clarity to ensure the elicitation of insightful responses from students. Forty-eight students provided written responses to the open-ended questionnaire. Given the language proficiency of the participants, the questions were designed in Chinese, students’ native language. Likewise, participants provided written responses in Chinese.

### 3.3 Data collection and analysis

This study complied with ethical principles in doing educational research (BERA, 2011). During the survey, participants were given instructions and sufficient time to respond to the questionnaires. For quantitative data, a total of 415 closed-ended questionnaires were obtained. Eight of them with missing values were excluded from further analysis. For qualitative data, 48 open-ended questionnaires were collected. The procedures of data analysis were as follows:

First, the missing values were checked, and the preliminary analyses (i.e., normality, Harman’s single-factor test, means, SD, and reliability analyses) were conducted. Then, three confirmatory factor analyses (CFA) were operated to test the validity of the measurements. Afterward, structural equation modeling (SEM) was conducted to explore the interplay of the constructs involved. All CFA and SEM analyses were conducted using Amos 27.0 with maximum likelihood estimation. Different indices (i.e., *χ*^2^/df, RMSEA, SRMR, CFI, TLI) were utilized to test the model fit. The chi-square to degrees of freedom ratio (*χ*^2^/df) should ideally fall between 1 and 3; the root mean square error of approximation (RMSEA) and the standardized root mean square residual (SRMR) are expected to be below 0.08; the comparative fit index (CFI) and the Tucker-Lewis index (TLI) should both exceed 0.90 (Bentler, 2007; Hu and Bentler, 1999).

The qualitative data were subjected to thematic analysis, a flexible method for systematically identifying, analyzing, and reporting meaning patterns in the raw data (Braun and Clarke, 2006; Miles et al., 2014). To begin with, the written responses underwent several close readings to ensure a thorough grasp of their meaning. The written responses were scrutinized line by line to extract meaningful units pertinent to participants’ perceptions of the interplay among teacher support, academic buoyancy, and L2 engagement. According to Gao and Zhang (2020), the open codes were first generated, such as “encouragement,” “care for me”, “word formation”, and “willing to invest time and effort in English learning”; then open codes were compared and grouped under axial codes, such as “emotional support”, “academic support”, and “L2 engagement.” Subsequently, axial codes were further abstracted into the sub-themes that capture patterns of the relationships, such as “academic support as a facilitator of L2 engagement”, “emotional support as a facilitator of academic buoyancy”, or “academic buoyancy as a facilitator of L2 engagement”. Next, the sub-themes were integrated into the overarching themes, including “teacher support as an influencing factor of L2 engagement” and “academic buoyancy as a mediator”. Last, several extracts were used to exemplify each theme and overarching themes, thereby substantiating the findings. To ensure the credibility of the coding process, the qualitative analysis was conducted by the first author and her colleague who published more than 10 articles in language teaching and learning, and the inter-coder reliability was checked using Krippendorff’s alpha (*α* = 0. 93). Discrepancies were resolved through discussion until consensus was reached. Member checking was used to improve trustworthiness, and four interviewees were asked to verify the extracted themes (Lincoln and Guba, 1985). Besides, an external scholar reviewed our analysis to ensure confirmability (Nassaji, 2020).

## 4 Results

### 4.1 The quantitative data

#### 4.1.1 Results of reliability and validity tests

The Harman’s single-factor test showed that the primary factor accounted for 42.449% of the total variance, falling below the 50% threshold suggested by Podsakoff et al. (2003). The test suggests that common method bias is unlikely to pose a serious threat to the validity of this study. Confirmatory factor analysis (CFA) was operated to assess the validity of the measurements used in the current study. The fit indices showed that each model had an excellent fit (see Table 1).

**Table 1:** The CFA fit indices (*N* = 407).

Constructs	*χ*^2^/df	RMSEA	SRMR	CFI	TLI
Teacher support	1.786	0.044	0.035	0.981	0.974
Academic buoyancy	1.098	0.016	0.007	1.000	0.999
L2 engagement	2.812	0.067	0.038	0.952	0.946

Table 2 showed the results for factor loadings, reliability, and convergent validity. All factor loadings exceeded 0.50. Internal consistency and convergent validity were assessed using Cronbach’s alpha and composite reliability (CR), along with average variance extracted (AVE). The criteria for acceptable values were Cronbach’s alpha ≥ 0.70, CR ≥ 0.70, and AVE ≥ 0.50 (Fornell and Larcker, 1981; Hair et al., 2009). As shown in Table 2, Cronbach’s alphas and CR values of all constructs exceeded 0.70, indicating good internal consistency and reliability. The AVE values for all three constructs exceeded 0.50 (teacher support = 0.586, academic buoyancy = 0.717, L2 engagement = 0.700), suggesting that most variance in the items reflected the intended latent constructs. Although the AVEs of the two dimension of teacher support (i.e., academic support and emotional support) were 0.456 and 0.483, slightly below the ideal threshold of 0.50, their CR values exceeded 0.70. According to Fornell and Larcker (1981, p. 46), the convergent validity can still be considered acceptable. Additionally, all CR values were higher than their corresponding AVE values, further supporting convergent validity (Hair et al., 2009).

**Table 2:** Results for factor loadings, reliability, and convergent validity *N* = 407.

Dimension/sub-dimension	Item	Unstandardized estimate	S. E	*Z*	*p*	Standardized estimate	Cronbach’s alpha	AVE	CR
Teacher support	Academic support	1.000				0.812	0.858	0.586	0.809
	Instrumental support	1.296	0.133	9.746	***	0.743			
	Emotional support	1.003	0.112	8.997	***	0.739			
Academic support	1	1.112	0.089	12.522	***	0.698	0.805	0.456	0.807
	2	0.931	0.079	11.776	***	0.653			
	3	1.313	0.108	12.158	***	0.676			
	4	1.182	0.098	12.047	***	0.669			
	5	1.000				0.679			
Instrumental support	1	1.018	0.063	16.109	***	0.858	0.830	0.624	0.832
	2	0.926	0.065	14.332	***	0.729			
	3	1.000				0.778			
Emotional support	1	1.026	0.092	11.134	***	0.724	0.741	0.483	0.736
	2	0.969	0.092	10.486	***	0.651			
	3	1.000				0.707			
Academic buoyancy	1	1.000				0.848	0.907	0.717	0.910
	2	1.117	0.050	22.149	***	0.859			
	3	1.050	0.049	21.279	***	0.839			
	4	1.116	0.052	21.349	***	0.841			
L2 Engagement	Agentic engagement	1.000				0.761	0.959	0.700	0.903
	Behavioral engagement	0.954	0.068	13.986	***	0.873			
	Emotional engagement	0.950	0.066	14.498	***	0.868			
	Cognitive engagement	1.007	0.075	13.352	***	0.840			
Agentic engagement	1	1.000				0.872	0.938	0.754	0.939
	2	1.005	0.043	23.329	***	0.855			
	3	1.017	0.046	22.258	***	0.834			
	4	1.111	0.044	25.383	***	0.892			
	5	1.107	0.044	25.250	***	0.890			
Behavior engagement	1	1.000				0.839	0.925	0.716	0.927
	2	0.971	0.047	20.724	***	0.832			
	3	0.848	0.044	19.219	***	0.793			
	4	1.093	0.047	23.057	***	0.887			
	5	0.990	0.044	22.608	***	0.877			
Emotional engagement	1	1.000				0.889	0.921	0.750	0.923
	2	1.028	0.045	22.763	***	0.835			
	3	1.087	0.043	25.108	***	0.877			
	4	1.104	0.046	24.202	***	0.861			
Cognitive engagement	1	1.000				0.816	0.930	0.628	0.931
	2	0.998	0.051	19.680	***	0.827			
	3	0.983	0.050	19.663	***	0.827			
	4	0.971	0.051	19.049	***	0.809			
	5	0.951	0.054	17.552	***	0.763			
	6	0.942	0.053	17.821	***	0.772			
	7	1.010	0.055	18.210	***	0.784			
	8	0.939	0.056	16.768	***	0.738			

***p < 0.001.

To assess discriminant validity, this study employed the Heterotrait-Monotrait (HTMT) ratio method proposed by Henseler et al. (2015, p. 121). As shown in Table 3, all HTMT values between latent variables were below the recommended threshold of 0.9, indicating adequate discriminant validity among the constructs. Taken together, these results demonstrate that the measurement instruments used in this study possess satisfactory reliability and validity, thereby meeting the prerequisites for subsequent data analysis.

**Table 3:** Results for HTMT *N* = 407.

Dimension	Teacher support	Academic buoyancy	L2 engagement
Teacher support			
Academic buoyancy	0.467		
L2 Engagement	0.577	0.829	

The HTMT results comes from SmartPLS.

#### 4.1.2 Descriptive statistics and correlation coefficients

As shown in Table 4, the absolute values of skewness (<2) and kurtosis (<7) are within the acceptable range according to West et al. (1995, p. 74). Hence, we may conclude that the data are in normal distribution and suitable for further analysis. Moreover, all components of L2 engagement are positively and significantly associated with those of teacher support as well as academic buoyancy (*r* = 0.200 ~ 0.695, *p* < 0.01).

**Table 4:** Descriptive statistics and correlation coefficients *N* = 407.

Variables	AS	IS	ES	AB	AE	BE	EE	CE
AS	1							
IS	0.574^**^	1						
ES	0.408^**^	0.355^**^	1					
AB	0.329^**^	0.302^**^	0.374^**^	1				
AE	0.242^**^	0.200^**^	0.445^**^	0.640^**^	1			
BE	0.387^**^	0.288^**^	0.433^**^	0.695^**^	0.576^**^	1		
EE	0.404^**^	0.323^**^	0.466^**^	0.638^**^	0.611^**^	0.410^**^	1	
CE	0.378^**^	0.338^**^	0.452^**^	0.671^**^	0.579^**^	0.664^**^	0.680^**^	1
Means	4.651	4.118	4.571	4.071	4.653	5.346	5.406	4.740
SD	0.689	0.852	0.712	0.927	1.082	0.845	0.912	0.919
Skewness	−0.356	0.073	−0.193	−0.335	−0.931	−0.971	−1.388	−0.566
Kurtosis	0.391	0.315	−0.262	0.310	1.520	2.799	4.150	1.430

**p < 0.01. AS refers to academic support; IS refers to instrumental support; ES refers to emotional support; AB refers to academic buoyancy; AE refers to agentic engagement; BE refers to behavioral engagement; EE refers to emotional engagement; CE refers to cognitive engagement.

#### 4.1.3 SEM analysis

**Figure 2:**
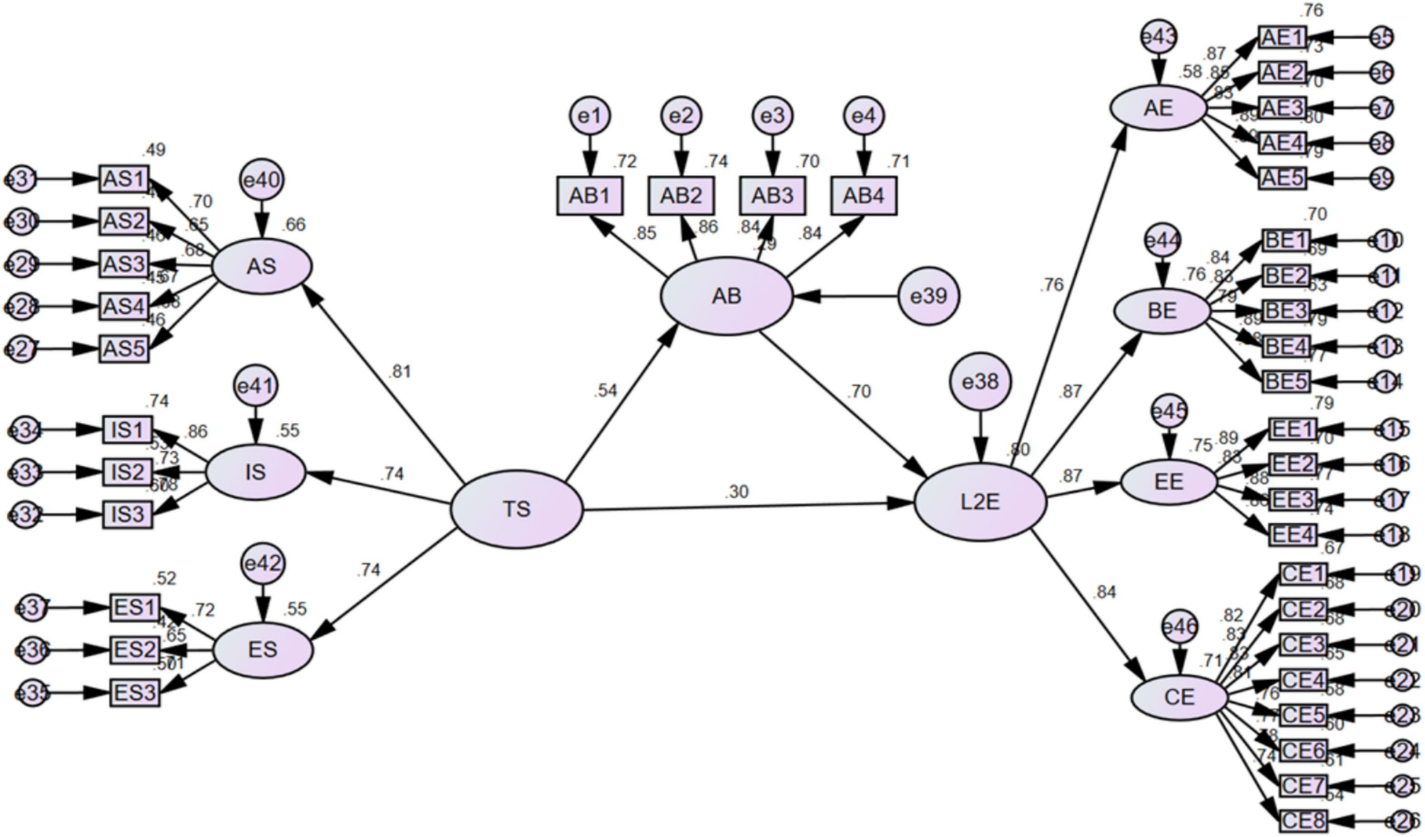
The structural model.

Using Amos 27.0, SEM was implemented to validate the hypothesized model. The model showed an excellent fit (*χ*^2^/df = 2.130; RMSEA = 0.053; SRMR = 0.038; CFI = 0.937; TLI = 0.932). The path coefficients were shown in Figure 2. According to Figure 2, teacher support has a significant direct influence on both academic buoyancy (*β* = 0.54, *p* < 0.001) and L2 engagement (*β* = 0.30, *p* < 0.001), while academic buoyancy emerged as a positive predictor of L2 engagement (*β* = 0.70, *p* < 0.001).

We subsequently tested whether academic buoyancy mediated the link between teacher support and L2 engagement by means of the Bootstrap method using 5,000 iterations. The details were shown in Table 5. According to Table 5, the 95% bias-corrected confidence interval for the indirect effect excluded zero (from 0.385 to 0.908), indicating that the path was significant (*β* = 0.567, *p* < 0.01). Besides, both the direct effect and the overall effect of the model were significant (*β* = 0.462, *p* < 0.01, CI = [0.210: 0.845]; *β* = 1.029, *p* < 0.01, CI = [0.695: 1.653], respectively). Thus, we may come to the conclusion that academic buoyancy functions as a partial mediator in the interplay between teacher support and L2 engagement. The second hypothesis was fully confirmed.

**Table 5:** The mediation analysis *N* = 407.

Pathway	β.	*p*	S. E.	95% CI	
				Lower	Upper
Indirect effect TS→AB→L2E	0.567	0.000	0.124	0.385	0.908
Direct effect TS→L2E	0.462	0.000	0.161	0.210	0.845
Total effect	1.029	0.000	0.234	0.695	1.653

TS refers to teacher support; AB refers to academic buoyancy; L2E refers to L2 engagement.

### 4.2 The qualitative results

The analysis of EFL learners’ perspectives on the interplay among L2 teacher support, academic buoyancy and L2 engagement yielded two overarching themes, namely teacher support as an influencing factor of L2 engagement and academic buoyancy as a mediator, illuminating the influence of teacher support on bolstering EFL learners’ academic buoyancy and L2 engagement.

#### 4.2.1 Teacher support as an influencing factor of L2 engagement

The positive influence of teacher support on EFL learners’ engagement was confirmed in the open-ended questionnaire. Forty-five students (93.8%) reported that teacher support could contribute to their L2 engagement. In total, the positive role of teacher support in enhancing EFL learners’ L2 engagement was mentioned 102 times in the written narrative data. Among its three dimensions, academic support (49 times) was identified as the most frequent influencing factor of L2 engagement; emotional support and instrumental support were also reported by students as the influencing factors of their engagement, 33 times and 20 times, respectively. Two excerpts were selected to demonstrate the influence of academic support on EFL learners’ L2 engagement:

P5: *My English teacher explains the knowledge very carefully during the class. I find English much easier, and I feel more engagement in learning it.*

P31: *My English teacher provides some specific guidance for my questions, and I am willing to be more engaged in learning English now*.

Besides, EFL learners also acknowledged the role of emotional support (e.g., encouragement, teacher care and praise) in increasing their L2 engagement. One excerpt was illustrated in the following:

P8: *The most useful guidance my English teacher gave me is the psychological help. I used to be afraid to speak English, but my English teacher often encourages me to speak English, and gradually I become less afraid and willing to express myself in English.*

EFL learners also emphasized the influence of instrumental support on their L2 engagement. For instance:

P23: *My English teacher’s recommendations of appropriate English learning materials make me more engaged in English learning*.

#### 4.2.2 Academic buoyancy as a mediator

Besides, 38 students (79.2%) reported that teacher support enhanced EFL learners’ resilience in overcoming difficulties and challenges in daily English learning, which in turn heightened their academic engagement. The role of teacher support in enhancing EFL learners’ academic buoyancy was mentioned 56 times in the written narrative data. Notably, emotional support (43 times) was identified as the most frequent theme to help them cope with setbacks and challenges during their English learning process. Two excerpts were illustrated as follows:

P19: *When I have difficulties in learning English, my English teacher’s encouragement and confirmation makes me feel that the difficulty is not a big deal. This kind of support makes me more willing to face challenges in my studies rather than giving up.*

P34: *My English teacher’s encouragement and support have boosted my confidence in addressing the challenges in English learning. I am able to engage myself in English learning with great effort.*

In addition, academic support and instrumental support from language teachers helped EFL learners to deal with the difficulties and problems in learning. Specifically, academic support and instrumental support were reported seven times and six times, respectively. Two excerpts were selected as follows:

P27: *I have difficulties in memorizing English words. My teacher told me that rote learning was not the only way to learn English words. She taught me to use roots and affixes to learn new words. I have been trying the method.*

P46: *My English teacher imparts useful learning methods and skills when I meet with difficulties and problems in language learning.*

All in all, the qualitative findings underscore that perceived teacher support serves as a critical catalyst for academic buoyancy among Chinese senior-high EFL learners, which in turn fosters greater engagement in language learning. The triangulation of the quantitative and qualitative results in the present research reveals that teacher support is quite significant in bolstering EFL learners’ academic buoyancy and enhancing their L2 engagement in learning tasks and activities.

## 5 Discussion

Drawing on the assumptions of the SDT, this research undertook a mixed-methods approach to examine the influence of student-perceived teacher support on enhancing L2 engagement in Chinese educational context. Moreover, the mediating role of academic buoyancy in the interplay between teacher support and L2 engagement was investigated. The quantitative analysis indicates a significantly positive relationship between EFL learners’ perceived teacher support and their L2 engagement, and academic buoyancy partially mediates this relationship.

First of all, SEM analysis shows there is a significant positive relationship between teacher support and L2 engagement. The first hypothesis is fully supported. In other words, EFL students tend to be actively involved in English learning when they perceive higher levels of support from English teachers, consistent with the findings of previous research (e.g., Chen, 2025; Sadoughi and Hejazi, 2021, 2023; Zhang and Wang, 2023). Compared with support from parents or classmates, teacher support has been shown to exert the strongest proximal effect on students’ active involvement in learning tasks (Wentzel et al., 2017) in that teacher support is more conducive to the fulfillment of students’ basic psychological needs (i.e., autonomy, competence, and relatedness). Specifically, teachers’ instrumental support may provide learners with choices in academic activities, thereby satisfying their need for autonomy. Students’ need for competence can be supported by teachers’ academic support (e.g., positive and constructive feedbacks; presenting optimal challenges), which “allows students to test and to expand their academic capabilities” (Niemiec and Ryan, 2009, p. 139). In the language classrooms, academic support may fulfill students’ need for competence by augmenting their linguistic proficiency (Tian and Zhou, 2020). Teachers’ emotional support may fulfill students’ need for relatedness (Derakhshan et al., 2025). When students’ basic psychological needs for autonomy, competence, and relatedness are supported in educational contexts, internalization of academic motivation will be facilitated and more autonomous engagement in learning activities will be promoted (Niemiec and Ryan, 2009).

Bootstrapped mediation estimates indicate that teacher support influences L2 engagement indirectly with academic buoyancy as a partial mediator. The second hypothesis is supported. To be specific, EFL learners’ perceived teacher support can significantly contribute to their academic buoyancy, congruent with extant literature in the field of general education (Chong et al., 2018; Granziera et al., 2022; Rohinsa et al., 2019) and in L2 settings (Derakhshan and Fathi, 2025; Fu, 2024; Li et al., 2025). That is to say, when EFL learners are provided higher levels of support from language teachers, they are equipped with more abilities to cope with setbacks and challenges in daily EFL learning. This finding could be accounted for by the facilitative role of teacher support in enhancing students’ self-regulatory capacities, which subsequently bolster their affective regulation, goal-setting skills, and capabilities to deal with setbacks and challenges (McEown and Sugita-McEown, 2019). When students perceive support from their language teachers, they are more likely to invest effort and maintain interest in language learning even if they encounter difficulties in students’ daily learning activities (Sadoughi and Hejazi, 2023). Hence, EFL teachers, as a pivotal factor in the practice of language learning, may provide learners considerable amount of support in order to foster their academic buoyancy.

Additionally, this study has found a significant relationship between academic buoyancy and EFL learners’ L2 engagement, a result that aligns with Wang (2024). This finding could be explained by the following reasons. First, buoyancy fosters positive emotions and self-efficacy beliefs, thereby strengthening emotional engagement (Xu and Wang, 2024). Second, it encourages planning behaviors that heighten behavioral engagement (Hirvonen et al., 2020). Third, it promotes the deployment of cognitive and self-regulatory strategies, which in turn boosts cognitive engagement (Collie et al., 2017; Xu and Wang, 2024). Last, academic buoyancy may stimulate EFL learners to be perseverant in making effort and sustain strong passion, and consequently become more active in language learning (Yang et al., 2022).

To obtain richer information about the interrelationships of the constructs under investigation, qualitative data are also collected through the open-ended questionnaire. The analysis of the qualitative data shows that student-perceived teacher support is a highly influential factor of academic buoyancy and L2 engagement, which is in accordance with the quantitative outcomes. As far as the specific facets of teacher support are concerned, academic support is identified as the most prominent factor in influencing Chinese secondary-level EFL learners’ L2 engagement. EFL learners are most eager to receive scholarly guidance and support from their language teachers to improve their linguistic competence. This may be explained by the educational context in China. Chinese secondary-level EFL learners often experience high academic pressure stemming from the very competitive national college-entrance examination that may exert a decisive influence on students’ future prospects (Hu and Schaufeli, 2009). EFL learners are eager to get more academic support from their language teachers in order to gain high scores in the college entrance examination. What merits special attention in the qualitative data is the pivotal role of emotional support in cultivating learners’ academic buoyancy. That is, teachers’ emotional support is very essential for EFL learners to cope with the difficulties in their regular language learning. Just as reported by the students, they are most eager to be cared and encouraged by their language teachers when they encounter setbacks and challenges in the daily English learning practice.

## 6 Conclusions, implications, limitations and directions for future research

Under the guidance of the SDT, the influence of teacher support on enhancing Chinese high school students’ L2 engagement is investigated in the current research. The study further probes whether academic buoyancy mediates the relationship between learners’ perceptions of teacher support and L2 engagement. The quantitative analysis demonstrates that student-perceived teacher support is positively associated with L2 engagement, and this association is partially mediated by academic buoyancy. The qualitative data have triangulated the above research findings. Based on these findings, we may conclude that EFL learners who perceive higher levels of support from teachers tend to equip with more abilities to cope with setbacks and challenges in daily English learning practice, and in turn they are more engaged in EFL learning. The current research underscores the crucial role of teacher support in the cultivation of academic buoyancy as well as the enhancement of L2 engagement in EFL contexts.

The present findings provide language instructors seeking to cultivate EFL learners’ academic buoyancy and enhance their L2 engagement with valuable insights. As the findings reveal, teacher support plays a significant role in cultivating EFL learners’ academic buoyancy and enhancing their L2 engagement. Among the three dimensions of instructor support, perceived academic support emerged as the most frequently reported influencing factor of L2 engagement in the EFL classroom, as revealed in the qualitative data. Academic support may therefore warrant greater pedagogical attention among language instructors, particularly within the Chinese educational context. Just as argued by Wang and Holcombe (2010, p. 655), “if teachers focus only on the social aspect but fail to attend to the academic aspect, students are less likely to be cognitively engaged in learning”. In addition, emotional support is the most frequently reported influencing factor of academic buoyancy in the EFL settings, as indicated in the qualitative data. Therefore, teachers should provide sufficient emotional support to EFL learners, such as caring about students and encouraging students to participate in classroom activities and expressing their opinions in a relaxed and enjoyable atmosphere (Liu et al., 2023; Ma, 2025). Besides, L2 teachers can support students instrumentally by sharing useful books, materials and some other learning resources (Liu and Li, 2023). For instance, language teachers can recommend various online resources for informal digital learning of English (IDLE), such as language learning apps, educational websites, and social media platforms, due to the fact that IDLE may boost L2 learners’ confidence, mitigate their negative emotions, and motivate them to be more engaged in learning a language (Zou et al., 2025b, 2026). Given the contributions of academic buoyancy to EFL learners’ engagement in language learning, language instructors should be aware of the necessities in cultivating learners’ academic buoyancy in EFL classrooms. Schools that support students’ basic psychological needs (i.e., autonomy, competence and relatedness) help reduce academic stress and refine their coping repertoires, thereby fostering academic buoyancy and strengthening their engagement in learning (Weinstein and Ryan, 2011).

There are several limitations to be admitted. To begin with, given the cross-sectional nature of the data, causal relationships between variables should be interpreted with caution. Second, as this study was conducted in China, the generalizability of the findings to other contexts remains uncertain. Third, only EFL learners participate in the present study, and future studies may survey or interview language teachers to acquire a nuanced grasp of the issue in question. Fourth, this study just explores the influence of teacher support on EFL learners’ academic buoyancy and L2 engagement. Future studies may investigate the influence of other teacher-related constructs, such as power use, classroom justice and authority, on EFL learners’ academic buoyancy and L2 engagement. Moreover, future studies are suggested to take ESL learners, instead of EFL learners, as the participants to examine the interrelationships of the variables under investigation.

## Data Availability

The original contributions presented in the study are included in the article/supplementary material, further inquiries can be directed to the corresponding author.
